# Rheology of transgenic switchgrass reveals practical aspects of biomass processing

**DOI:** 10.1186/s13068-018-1056-5

**Published:** 2018-03-01

**Authors:** Guigui Wan, Taylor Frazier, Julianne Jorgensen, Bingyu Zhao, Charles E. Frazier

**Affiliations:** 1 Sustainable Biomaterials, Virginia Tech, 230 Cheatham Hall, Blacksburg, VA 24061 USA; 2 Macromolecules Innovation Institute, Virginia Tech, Blacksburg, VA 24061 USA; 3 Horticulture, Virginia Tech, 407 Latham Hall, Blacksburg, VA 24061 USA; 4 Franklin W. Olin College of Engineering, 1000 Olin Way, Needham, MA 02492 USA

**Keywords:** Glass transition, Lignin, Rheology, Switchgrass (*Panicum virgatum*), Transgenic

## Abstract

**Background:**

Mechanical properties of transgenic switchgrass have practical implications for biorefinery technologies. Presented are fundamentals for simple (thermo)mechanical measurements of genetically transformed switchgrass. Experimental basics are provided for the novice, where the intention is to promote collaboration between plant biologists and materials scientists.

**Results:**

Stem sections were subjected to two stress modes: (1) torsional oscillation in the linear response region, and (2) unidirectional torsion to failure. Specimens were analyzed while submerged/saturated in ethylene glycol, simulating natural hydration and allowing experimental temperatures above 100 °C for an improved view of the lignin glass transition. Down-regulation of the 4-Coumarate:coenzyme A ligase gene (reduced lignin content and altered monomer composition) generally resulted in less stiff and weaker stems. These observations were associated with a reduction in the temperature and activation energy of the lignin glass transition, but surprisingly with no difference in the breadth and intensity of the tan *δ* signal. The results showed promise in further investigations of how rheological methods relate to stem lignin content, composition, and functional properties in the field and in bioprocessing.

**Conclusions:**

Measurements such as these are complicated by small specimen size; however, torsional rheometers (relatively common in polymer laboratories) are well suited for this task. As opposed to the expense and complication of relative humidity control, solvent-submersion rheological methods effectively reveal fundamental structure/property relationships in plant tissues. Demonstrated are low-strain linear methods, and also nonlinear yield and failure analysis; the latter is very uncommon for typical rheological equipment.

## Background

Switchgrass (*Panicum virgatum*), a perennial warm season grass native to North America, has been identified as important for development into an herbaceous biomass fuel crop [1–3]. Such crops should efficiently release sugars from cell wall polysaccharides through (bio)chemical conversion. Lignin has complex associations with polysaccharides, and its presence limits the accessibility of plant cell wall polysaccharides to chemical, enzymatic and microbial digestion [1–3]. The lignin content of feedstocks has been proposed as one key agronomic trait affecting biofuel production from lignocellulosic biomass. To improve biomass fermentability, genetic modifications aiming to lower lignin content and modify lignin structure have been extensively developed [4–7]. Besides sugar accessibility, such modifications could impact plant viability as well as the practical aspects of biomass handling and processing [1, 5, 8]. In light of these issues, it is appropriate to develop rheological methods that reveal how plant tissue mechanical properties are affected in genetically modified switchgrass with reduced lignin content.

Rheology is the study of deformation and flow in materials, both liquids and solids. Carefully measured deformations reveal insights about molecular structure, hierarchical organization, and aspects of material processing [9]. Dynamic mechanical analysis (DMA) is one common rheological method involving application of an oscillating stress or strain, and measurement of the resulting strain or stress. The DMA response in polymeric materials is viscoelastic; it is simultaneously elastic like a spring, and viscous like flowing water. However, in viscoelastic solids, the viscous response is not macroscopic liquid flow, but rather localized flow as when lignin segments rub and slip past one another in dissipation of mechanical energy. If carefully conducted, the DMA of lignocellulose may be used to reveal insights about the structure and organization of cellulose, hemicelluloses, and lignin. Most of this type of research has been devoted to wood, with examples such as [10–14]. Given the variety of polymers and the complexity of their organization in lignocellulose, it is interesting to note that lignocellulose exhibits only one major thermomechanical softening transition, a glass-to-rubber transition attributed to lignin [14]. The in situ lignin glass transition is quite sensitive to moisture levels, meaning that moisture control is a very practical experimental concern [10]. In the present work, switchgrass specimens were analyzed while immersed/saturated in ethylene glycol. This approach simulates natural hydration in the living plant, provides a broader experimental temperature range (compared to water), and is quite simple in comparison to relative humidity control [12]. Chowdhury et al. demonstrated that the thermorheological response of switchgrass stem is quite similar to that of wood, and tissue maturity effects are easily detected [12]. The objectives of this paper are to demonstrate solvent-submersion rheological methods that reveal whole-tissue mechanical properties that reflect genetic lignin modifications, and to provide experimental details that help biologists collaborate with materials scientists. The intention is to address practical experimental challenges that help those interested in biomass rheology. Others have studied the rheological properties of transgenic lignocellulose using tension or bending stress modes [15–18]. We suggest that the torsional stress mode (torsional axis parallel to the plant stem, and specimen clamped under a minor tensile stress) offers advantages for the analysis of small lignocellulose samples. This so-called tensile–torsion analysis was discussed in [12]. In the work described here, switchgrass specimens were subjected to DMA that was carefully restricted to the linear viscoelastic region. Additionally, a novel unidirectional continuous stress-ramp was imposed such that switchgrass stem sections underwent yielding and failure. The properties measured could have implications for plant viability in the field, and for aspects of biomass processing.

## Methods

### Selection of RNAi:4CL transgenic switchgrass plants

**Fig. 1:**
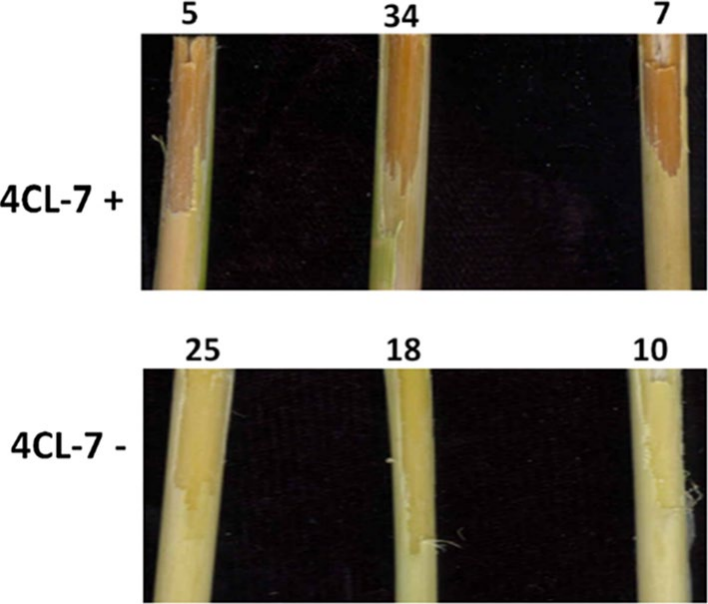
Stem color phenotype in the *RNAi:Pv4CL1* transgenic positive (+, top) and negative (−, bottom) plant specimens. The presence of the *RNAi:Pv4CL1* transgene correlated with brown stem color (top), while transgene absence correlated with green stem color (bottom). Plant 40(+) exhibited brown stem color as other positive plants

In a previous study, the T_0_ transgenic switchgrass plants expressing an *RNAi:Pv4CL1* construct with reduced lignin content were characterized [7]. One *RNAi:Pv4CL1* transgenic line 4CL-7 was selected to cross with the wild-type switchgrass plants (HR8). The derived hybrid plants (T_1_) with or without the transgene *RNAi:Pv4CL1* were identified. The T_1_ plants carrying the *RNAi:Pv4CL1* resulted in silencing of the *Pv4CL1* gene, which causes a brown plant stem color, while T_1_ plants without the transgene will have green stems (Fig. 1). Seven T_1_ plants derived from the 4CL-7 plants that have been confirmed by PCR analysis (data not shown) were selected for this study: three plants (10−, 18−, 25−) where the transgene was absent (green stem), indicated by the negative sign (−), and four plants (5+, 7+, 34+, 40+) where the transgene was present (brown stem), indicated by the positive sign (+). The plants were maintained in a greenhouse under a 16-h day/8-h night photoperiod. The lignin in these samples was not analyzed, but because the reddish pigment accumulated in the stem of *RNAi:Pv4CL1* plants is co-related with the reduced lignin content, it is assumed the T_1_ transgenic lines have reduced lignin content as previously described [7]. Consequently, the transgene negative plants in this study are similar to wild-type plants (H/G/S, respectively, *p*-hydroxyphenyl, guaicyl, and syringyl, monomer ratio = 0.04/1.05/1.00); whereas, the transgene positive plants have about 22% less lignin, with an altered H/G/S monomer ratio = 0.07/0.56/1.00.

### Tissue collection and stem sample preparation

Tillers with an elongation stage of four internodes were freshly harvested from all switchgrass plants at soil level and then divided into different parts according to the location of the internodes [19]. The internode closest to the soil was labeled as the first internode. The second internode, which is positioned adjacent to the first one and second from soil level, was selected for analysis. Within the second internode, the stem was sectioned into small pieces with lengths of 2–3 cm. The 2- to 3-cm-long sections (hollow cylinders) were cut along the stem length and subsequently cut into rectangular shapes with widths between 3 and 5 mm. The thicknesses of the specimens ranged from 0.7 to 1.0 mm. For each plant specimen, at least three tillers (second internode) were obtained from three individual plants, and rectangular sections from a single plant were randomized into one batch. After rectangular sections were prepared, they were immediately transferred into ethylene glycol, vacuum-treated (20 Torr) for 30 min, followed by vacuum release and solvent immersion at atmospheric pressure for at least 48 h. After this solvent saturation, all specimens were stored at 12 °C.

### Rheological setup

**Fig. 2:**
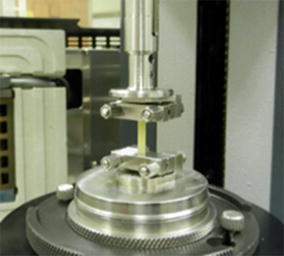
Specimen clamping system assembled without the surrounding solvent-submersion hardware

Specimens were subjected to solvent-submersion analysis in torsional stress mode using a TA Instruments AR-G2 rheometer with a concentric cylinder attachment containing a fluid-cooled jacket. Specimens were secured in tension clamps (40 cN × m torque) where the specimen long axis was parallel to the torsional axis, Fig. 2. Other relevant details are found in [12]. During all tests, specimens were subjected to 1 N static tensile force and immersed in ethylene glycol. Specimen length between the clamps was 0.8–1.5 cm, and the thickness and width dimensions were averaged from three locations within this length dimension.

### LVR determination

Great care was exerted to ensure that dynamic (oscillatory) experiments were conducted within the linear viscoelastic response (LVR). More elaboration appears later, but the LVR is the low-strain response region where the stress and strain exhibit a linear relationship. At much higher strains, the stress/strain response becomes nonlinear. The LVR is typically defined using frequency sweep experiments (fixed stress applied over a range of oscillation frequencies) and stress sweep experiments (fixed oscillation frequency applied over a range of stress). In the present case, this typically involved numerous frequency sweep and stress sweep experiments conducted (on sacrificial specimens) at the temperature extremes, 25 and 120 °C. Frequency sweeps were typically conducted from 0.01 to 10 Hz; stress sweeps were typically conducted from 5000 to 60,000 Pa. Dynamic stress/strain plots were fitted to a line and the LVR stress limit was defined as the stress beyond which the correlation coefficient (*R*^2^ for the least squares fit) was less than 0.99995.

### Temperature ramps

Once typical LVR behavior was established, separate specimens were subjected to temperature ramp experiments as follows: (1) equilibrate, 25 °C, 5 min, (2) stress sweep, 5000–50,000 Pa, 0.5 Hz, 25 °C, (3) heat, 25–120, 2 °C/min, 0.5 Hz, 50,000 Pa, (4) equilibrate, 120 °C, 5 min, (5) stress sweep, 5000–50,000 Pa, 0.5 Hz, 120 °C, (6) cool, 120–25, 2 °C/min, 0.5 Hz, 50,000 Pa. Stress sweeps were conducted at the temperature extremes before and after the temperature ramps to further verify that all analyses were conducted within the LVR.

### Time–temperature superposition (TTS)

TTS experiments were conducted as a series of isothermal frequency sweeps as follows: (1) rapidly heat to 120 °C; equilibrate 5 min, (2) cool, 120–25, 2 °C/min, 50,000 Pa, 0.5 Hz, (3) rapidly heat to 120 °C; equilibrate 5 min, (4) isothermal frequency sweep, 0.05–0.5 Hz, 50,000 Pa, 120 °C, (5) reduce temperature by 5 °C; equilibrate 5 min; isothermal frequency sweep, 0.05–0.5 Hz, 50,000 Pa, (6) repeat step 5 to obtain isothermal frequency sweeps from 110 to 25 °C, (7) rapidly heat to 120 °C; equilibrate 5 min, (8) cool, 120–25, 2 °C/min, 50,000 Pa, 0.5 Hz. Note that cooling ramps were conducted before and after the sequential isothermal frequency sweeps as a means to determine if any substantial thermal degradation occurred during this analysis.

### Torsional shear strength

Unidirectional torsional stress ramps were conducted until specimen failure. During analysis, the specimen was clamped in a similar fashion as above, but without solvent submersion (specimens were saturated in ethylene glycol as above). All experiments were conducted at ambient temperature (~ 22 °C). The AR-G2 rheometer was operated in continuous unidirectional displacement with a shear stress increasing from 100,000 to 100,000,000 Pa, using linear mode data acquisition over a 33-min period collecting 300 data points.

### Statistical analysis

JMP^®^ software was employed for pair-wise non-parametric Wilcoxon analysis.

## Results and discussion

In DMA, a specimen is subjected to an oscillatory stimulus (stress or strain) and the corresponding mechanical response (strain or stress) is measured. In plant tissues and other viscoelastic materials, the oscillatory response contains two components: the storage modulus and the loss modulus. The former relates to elastic energy storage as in a Hookean spring, and the latter deals with viscous energy dissipation as in a dashpot filled with a simple Newtonian liquid [20, 21]. A principal concern in DMA is verified operation within the linear viscoelastic response (LVR), the low-strain region where stress and strain are linearly related, where results are independent of input levels (stress or strain) and mathematical descriptions of the raw data are well understood [20–22]. Operating within the LVR allows an accurate accounting of energy storage and energy loss phenomena upon which structural models and structure/property relationships are confidently developed.

**Fig. 3:**
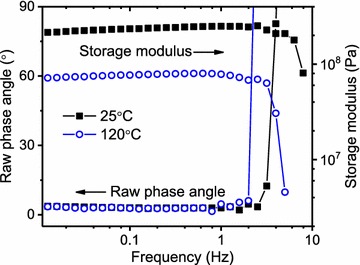
Example frequency sweep at temperature extremes, 25 °C (black square) and 120 °C (blue circle), for a specimen (4CL-7 derived line 40+) immersed in ethylene glycol; oscillation stress = 50,000 Pa

**Fig. 4:**
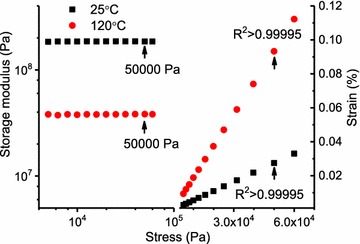
Typical stress sweep data (right) used to establish the LVR limit at the temperature extremes, 25 °C (black square) and 120 °C (red circle) (specimen: 4CL-7 derived line 5+; 0.5 Hz). Arrows (storage modulus, left) indicate maximum stress used in the linear stress/strain fit

Another practical concern is specimen size. Oftentimes, genetically transformed plant tissues are only available in very small sizes, and this has implications for the type of rheological equipment used. In our experience, rotational rheometers are preferred for the DMA of plant tissues [12], whereas many other polymer scientists prefer bending and/or tensile-mode DMA equipment. When the torsional axis is parallel to the plant stem, as in this case, torsional stress engages the amorphous matrix to a greater degree than tension or bending along the plant stem, where cellulose fibrils dominate the response [17]. Polymer laboratories often possess rotational rheometers, and most common are stress-controlled machines because they are less expensive than strain-controlled rheometers. When using small, low-stiffness specimens with a stress-controlled rheometer, as in this work, caution is required to avoid inertia effects where the specimen stiffness is overwhelmed by the momentum of the specimen clamping system. Consequently, frequency sweep experiments (fixed stress applied over a range of oscillation frequencies) are required to determine valid oscillation frequencies that satisfy LVR criteria. Figure 3 demonstrates typical data from this work. At oscillation frequencies above 2 Hz, the storage moduli exhibited a sharp decline, and the raw phase angles (phase lag between input stress and measured strain) showed a correspondingly abrupt increase to values exceeding the theoretical maximum of 90°. Figure 3 indicates that inertia effects prevent operation at oscillation frequencies above 2 Hz. However considering biomass variability, the maximum oscillation frequency in this work was limited to 0.5 Hz. Having established the maximum oscillation frequency, the maximum stress levels were determined with a stress sweep (fixed oscillation frequency applied over a range of stress) at the frequency maximum, as shown in Fig. 4. Note that the determination of maximum stress and frequency settings is a “chicken or egg” exercise where the investigator asks, “Which parameter is first optimized?” Since stress and frequency effects are coupled, the investigator must conduct iterations between stress and frequency adjustments until the desired settings are approached. In other words, it is not as simple a matter as the progression from Figs. 3 to 4 might suggest. A careful and deliberate development of acquisition parameters is warranted, and preferably using sacrificial specimens that might experience irreversible changes if preliminary experiments grossly exceed the LVR.

The LVR limit was arbitrarily defined as the maximum stress in which a linear fit to the stress/strain plot provided a correlation coefficient (*R*^2^) not less than 0.99995. Figure 4 demonstrates that this criterion was very conservative; in other words for this specimen, a safely linear response extended beyond the LVR limit defined here. The maximum oscillation frequency (0.5 Hz) and maximum oscillation stress (50,000 Pa) settings used in this work were conservatively restricted to assure operation within the LVR and therefore provide confidence in data interpretation among all plants studied. Others might safely select less conservative acquisition parameters. As a practical matter, DMA signal-to-noise tends to increase with increasing stress levels. Consequently, one would wish to operate at the highest possible stress level that remains within the LVR. However, the LVR behavior of biomass can be highly variable as demonstrated in *Liriodendron tulipifera* wood by [23]. Consequently, we elected to remain conservatively within the LVR and at times this resulted in poor signal-to-noise, as will be discussed later.

**Fig. 5:**
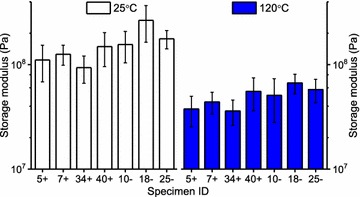
Average storage moduli of all switchgrass specimens at 25 and 120 °C. Obtained from stress sweep experiments; number of observations *n* = 5–6; error bars = ± 1 standard deviation

Figure 5 compares the storage moduli (dynamic stiffness) of all specimens at the temperature extremes used in this work. These storage moduli were obtained from stress sweeps before and after the temperature ramps discussed below. The low- and high-temperature storage moduli are often referred to respectively as the “glassy” and “rubbery” moduli (glassy implies stiff; rubbery means soft). However, because specimens were saturated/immersed in ethylene glycol, the glassy (low temperature) moduli shown in Fig. 5 are not as high as commonly observed for synthetic polymer glasses (which are ~ 10^9^ Pa). As is typical, the rubbery moduli (high temperature) are much lower, and this temperature-induced softening is attributed to the lignin glass transition [13, 14]. However, it is not entirely clear if and how hemicelluloses impact this transition. As measured in ethylene glycol, this softening behavior is similar in appearance to that observed in water, but that a more nearly complete softening transition is observed in the higher boiling ethylene glycol [12]. The magnitude of softening shown is less than one decade (factor of 10) of storage modulus, and this is within the typical range for liquid-saturated lignocellulose (~ 0.6–1 decade). Relative to non-filled thermoplastic polymers, this is a minor degree of softening, reflective of a low volume fraction matrix dispersed among highly oriented cellulose fibrils.

Within transgene positive and negative groups, the samples were nearly all of the same stiffness at the respective temperatures, Fig. 5. After pooling the data within transgene groups, Table 1 shows that plants containing the *RNAi:Pv4CL1* transgene (positive samples) were less stiff than the non-transgenic controls (negative samples). Considering the 25 °C data, this implies that on average the plants expressing *RNAi:Pv4CL1* might be more or less resistant to wind loading and lodging effects—however, such effects were not studied. On the other hand, transgene positive plants may require less energy for milling and other physical pretreatments. At high temperature, the reduced stiffness of transgene positive plants is very possibly associated with easier lignin removal and greater access to cellulose, as discussed later.

**Table 1:** Probability values (*p* values) from the transgene (+) and the transgene (−) pooled intergroup comparison of storage moduli (25 and 120 °C); number of observations = 20–25

	Storage moduli	
	25 °C	120 °C
*p* value (+) vs (−)	0.001	0.007

**Fig. 6:**
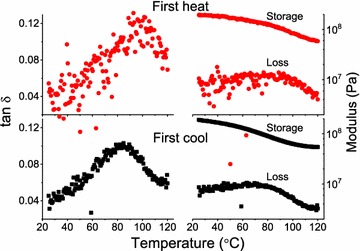
Example first heat (red circle) and first cool (black square) of a switchgrass specimen (the 4CL-7 derived line 10−) immersed in ethylene glycol, stress setting at 50,000 Pa, frequency 0.5 Hz

**Fig. 7:**
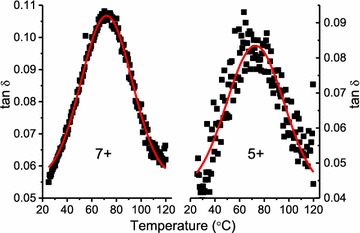
Examples of good (left) and poor (right) first cool tan δ curves, and the nonlinear fitting (red) used to improve precision in the *T*_g_ (temperature at tan δ maximum) determination

Figure 6 presents typical first heat and first cool temperature ramps of a switchgrass specimen (10−) immersed in ethylene glycol. Shown are the storage modulus, described above, and the tan *δ* which is the simple ratio of loss modulus to storage modulus (indicative of mechanical energy absorption). Typically, the first heat scan was noisy in the loss modulus signal, which is magnified in the tan *δ*. The noisy response is attributed to the low-stress settings (50,000 Pa) that were selected to assure that analysis remained within the LVR. Characteristically for these samples, the subsequent first cool exhibited much less noise. Since the first heat and first cool acquisition parameters were identical, the improved signal quality in the first cool perhaps reflects specimen stress relaxation (mechanical conditioning) within the clamping mechanism, leading to an improved signal. First cool scans were not always ideal; for instance, best and worst case examples are presented in Fig. 7. Because of the signal noise demonstrated, first heat glass transition temperature (*T*_g_, temperature at tan *δ* maximum) data are omitted here, and all first cool *T*_g_ were identified using nonlinear curve fitting of the first cool tan *δ* curve. Curve fitting increases the precision of *T*_g_ determination, while also improving estimates of the tan *δ* maxima (tan *δ* max), and the width of the tan *δ* signal at half-maximum (width). Table 2 presents pooled data and demonstrates that transgene positive plants exhibited *T*_g_ about 11 °C lower than for the transgene negative samples. The lignin in these specimens was not analyzed, but it is assumed to be similar to the T_0_ transgenic lines described previously [7]. Consequently, the transgene negative plants in this study are similar to wild-type plants with an H/G/S monomer ratio = 0.04/1.05/1.00) (H = *p*-hydroxyphenyl unit; G = guaicyl unit; and S = syringyl unit); whereas the transgene positive plants had about 22% less lignin, with an altered H/G/S monomer ratio = 0.07/0.56/1.00 [7]. Having a substantially reduced quantity of G-monomers, the lignin in the transgene positive plants was more flexible, either because of less lignin crosslinking, and/or because of the greater free volume associated with the dominant S-monomers [14]. These effects explain why a related population of transgene positive plants yielded more fermentable sugar for biofuel production [7].

**Table 2:** Average glass transition (*T*_g_), tan *δ* maxima (tan *δ* max), and width of the tan *δ* signal at half-maximum (width) for pooled (+) and (−) samples (determined through curve fitting as described); number of measurements *n* = 9–12, standard deviations in parentheses

	*T*_g_	tan *δ* max	Width
(−)	78.9 (2.7)	0.10 (0.02)	21.1 (1.5)
(+)	67.8 (3.0)	0.09 (0.01)	21.3 (2.1)
*p* value (+) vs (−)	< 0.001	0.20	0.81

**Fig. 8:**
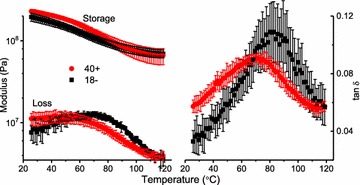
Comparison of first cool scans for the specimen of 4CL-7 derived line 40+ (red circle) and line 18− (black square) specimens; stress setting at 50,000 Pa, frequency 0.5 Hz, number of observations, *n* = 3. Error bars = ± 1 standard deviation

On the other hand, it is not entirely clear why the tan *δ* maxima (tan *δ* max), and width of the tan *δ* signal at half-maximum (width), were not significantly different between the transgene groups, Table 2. Roughly speaking, these parameters reflect the quantity of relaxing polymer (tan *δ* max) and the heterogeneity of lignin relaxation (width). Given a 22% difference in lignin content, one might expect the tan *δ* max and width parameters to differ between the transgene positive and negative specimens. Perhaps this indicates that the tan *δ* max and width parameters reveal less about lignin relaxation and more about the lignin/polysaccharide covalent attachments, as in the LCC (lignin-carbohydrate complex) [24, 25]. A general comparison of the transgene groups is provided in Fig. 8, where the 40+ and 18− specimens were selected. Notable here is that the rubbery moduli did not vary between these sample types, but recall that the rubbery moduli were significantly different according to the pooled data set (Table 1). A related observation was made by Olsson and Salmén where chemically induced lignin crosslinking increased the *T*_g_ in spruce wood, but with no impact on the rubbery storage modulus [26]. In synthetic network polymers, increased crosslinking normally raises the *T*_g_ and this is typically accompanied with an increased rubbery storage modulus [20]. Currently, it is unknown if and how the rubbery modulus is affected by the LCC [24, 25]. Not addressed in this work are possible changes in bulk tissue density, as found in transgenic *Populus* exhibiting a large reduction in lignin content [17]. Changes in bulk density should be addressed in more detailed studies, in addition to microfibril angle measurements [27].

**Fig. 9:**
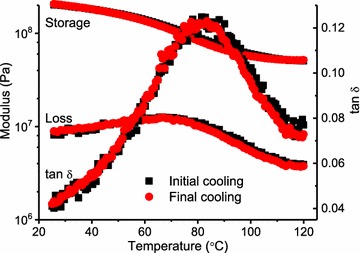
Example cooling curves before (black square) and after (red circle) TTS isothermal frequency scans for the specimen of 4CL-7 derived line 18−

A more detailed analysis of the lignin glass transition is available through time–temperature superposition (TTS); 40+ and 18− were selected to represent each transgene group. TTS procedures place the specimens under prolonged heating, and the extent of possible thermal degradation should be determined by comparing simple temperature ramps before and after the TTS procedure. Figure 9 demonstrates that the TTS procedure caused little or no thermal degradation in the switchgrass specimens.

**Fig. 10:**
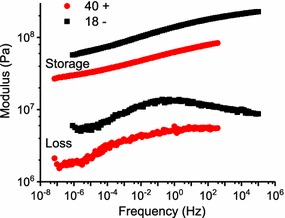
Example storage and loss modulus master curves for the specimen of 4CL-7 derived line 40+ (red circle) and line 18− (black square) specimens

**Fig. 11:**
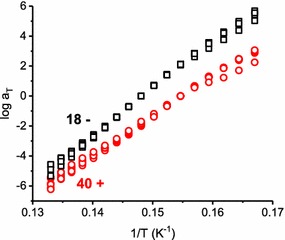
Shift factors for the specimen of 4CL-7 derived line 18− (black square; *n* = 4) and line 40+ (red circle; *n* = 6)

For TTS analysis, isothermal frequency sweeps were collected in 5 °C temperature increments (in cooling fashion) across the glass transition. The separate frequency sweeps were shifted on the frequency axis such that a smooth “master curve” resulted [13]. This master curve simulates the rheological response over a much broader frequency range, as for the examples in Fig. 10. Notable is that the storage modulus master curves were very smooth, which is typical; and the loss modulus curves were less perfect but still quite smooth. This is unusual for lignocellulose; oftentimes, the loss modulus master curves are much less smooth than those in Fig. 10 [13]. The smoothness, or lack thereof, in lignocellulose master curves might reflect aspects of supramolecular organization that promote thermorheological complexity in lignocellulose [13]. When creating the master curves, the so-called “shift factor” (a_T_) records the magnitude of the frequency shift and the corresponding shift factor plot reflects the temperature dependence of the lignin relaxation, Fig. 11.

The shift factor plot allows for determination of the activation energy associated with the glass transition (*E*_a_) via the Arrhenius equation [28]:

$$\log a_{\text{T}} \, = \,\frac{{E_{\text{a}} }}{2.303\, \times \,R}\left( {\frac{1}{T}\, - \,\frac{1}{{T_{\text{o}} }}} \right)$$

*R* is the gas constant and *T*_o_ is the reference temperature in K.

Table 3 summarizes *E*_a_ for samples 40+ and 18− calculated using the equation above. 40+ exhibited an *E*_a_ of 261 kJ/mol, significantly lower than for 18− (311 kJ/mol). These activation energies are within the range reported for wood, where higher activation energies are associated with higher *T*_g_ [13, 26]. As stated before, the thermomechanical softening of lignocellulose is related to localized motions of lignin segments. Therefore, the activation energy associated with the glass transition should reflect the content and composition of lignin. The transgene positive plant had substantially less lignin and G monomer, relative to the negative plant, and this lead to less lignin crosslinking, greater flexibility and therefore reduction in the temperature and activation energy of the lignin glass transition. As previously mentioned, these effects likely explain why transgene positive plants yielded more fermentable sugar for biofuel production [7].

**Table 3:** Activation energy associated with the glass transition for specimens 40+ (*n* = 6) and 18− (*n* = 4)

4CL-7	Activation energy (kJ/mol)
18−	311 (15)
40+	261 (15)

Standard deviation in parentheses

**Fig. 12:**
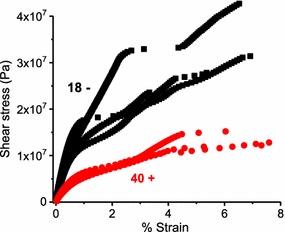
Torsional stress/strain response of 40+ (red circle; *n* = 3) and 18− (black square; *n* = 4) specimens saturated in ethylene glycol

The prior discussions addressed dynamic loading conducted at very low-stress levels within the LVR. Equally important is the mechanical response at high, nonlinear stress levels where yielding and failure occur. Such strength testing is typically excluded from the highly sensitive equipment used for polymer rheology. However, the torsional stress mode can accommodate high-strain levels, and the small switchgrass samples are sufficiently weak to provide the unusual opportunity to conduct strength testing using the torsional rheometer. In this case, a unidirectional torsional stress sweep was applied to transgene positive and negative samples until failure occurred. Of the two sample types tested, Fig. 12 demonstrates that transgene positive samples were clearly less stiff (as previously demonstrated) and substantially weaker than transgene negative samples. Table 4 lists the yield stress (nonlinear onset) and shear strength (maximum stress) corresponding to Fig. 12. Note that the torsional strength of wood may be estimated as equivalent to shear strength parallel to grain [29]. Liu and Koc found that the shear strength, parallel to the stem, for switchgrass was about 2 MPa [30], or about 10–30 times less than the average torsional strength reported here.

**Table 4:** Yield stress and shear strength for samples 18− (*n* = 4) and 40+ (*n* = 3)

	Yield stress (Pa)	Shear strength (Pa)
18−	6.35 × 10^6^	3.14 × 10^7^
	(1.53 × 10^6^)	(8.13 × 10^6^)
40+	1.67 × 10^6^	1.28 × 10^7^
	(6.17 × 10^5^)	(1.88 × 10^6^)

Standard deviation in parentheses

## Discussion

As mentioned, *RNAi:Pv4CL1* transgene positive plants were previously shown to yield more fermentable sugar, and this was attributed to a reduced content and altered monomer composition of lignin [7]. The corresponding thermomechanical effects were detected as reductions in stiffness, strength, and also as reductions in the temperature and activation energy of the lignin glass transition. Surprisingly, the intensity (tan *δ* max) and breadth (width) of the glass transition in transgene positive and negative plants were not significantly different, perhaps because the RNAi:Pv4CL1 transgene did not affect the number of lignin/polysaccharide covalent attachments. This hypothesis requires testing, but any hypothesis must be grounded upon rigorous satisfaction of the LVR criterion where simple Hookean springs and Newtonian dashpots can be confidently equated to hypothetical molecular features of the cell wall. Repeated emphasis of the LVR criterion is perhaps tiresome for experienced rheologists, but this point is rarely emphasized in the literature. More importantly, the lesson is invaluable for beginners, and for collaborations among materials scientists and plant biologists, who should remind the rheologist that LVR behavior in plant tissues is highly variable [23].

Even experienced rheologists will appreciate the novelty of specimen yielding and failure analysis (strength testing) conducted here (Fig. 12), and this speaks to the unique suitability of rotational rheometers for the analysis of small plant tissues. The torsional stress mode has an unlimited strain capacity, and the small switchgrass specimens were weak enough to load through yielding and failure. Combined with linear DMA, this nonlinear strength testing provides much more information from one simple approach. Furthermore, a quick change to the parallel-plate geometry allows one to analyze specimens that lack mechanical integrity, such as fibrous mats resulting from biomass pretreatment [12]. Stress-controlled torsional rheometers are also easily fashioned with solvent-submersion capabilities, whereas traditional bending/tensile-mode DMA machines require more specialized equipment. Solvent-submersion analysis is one convenient way to address the need for maintaining specimen moisture (or plasticizer) control. Whereas relative humidity control is more expensive and less convenient, but absolutely required for certain purposes.

The examples presented herein demonstrate that lignin alterations (that improve glucose accessibility) are associated with changes in the lignin glass transition, as well as reductions in tissue stiffness and strength. These effects could possibly reduce energy requirements for milling plant tissues prior to chemical pretreatments. The corresponding impact on lodging and plant viability is unknown. However, antisense 4CL poplar (*Populus* sp.) lines exhibited wood with reduced stiffness and strength; and field-grown plants that were not staked were phenotypically smaller than wild-type plants [31]. Obviously, the relationships between rheological behavior determined here and the actual impacts on plant viability and energy requirements for biomass milling need to be determined. Touched upon here are lignocellulose structure/property relationships that will impact biorefinery technologies, and the need to refine these methods and actually correlate them to practical concerns is apparent. Once these correlations are established, we believe that these rheological methods could help accelerate the development of biorefinery technologies.

## Conclusions

Rheology of plant tissues is extremely valuable to help molecular biologists understand the thermomechanical effects resulting from the genetic transformations they devise. In this example, down-regulation of Pv4CL1 caused lignin modifications that increased fermentable sugar yields; and this was associated with reductions in the lignin glass transition temperature and activation energy, as well as reductions in plant stem stiffness and strength. Fundamental experimental details were outlined to benefit newcomers to plant tissue rheology, and to help plant biologists master the simple yet critical questions they should ask when collaborating with polymer rheologists. Data quality and rigorous adherence to linear viscoelastic analysis were emphasized so that plant cell wall molecular models are created on a sound theoretical foundation. A novel nonlinear analysis was demonstrated that provides yielding and failure data (strength testing) that is atypical for highly sensitive rheological equipment. This novelty was attributed to the torsional rheometer, and a strong case was made for the unique suitability of the torsional rheometer for rheology of very small plant tissues.
